# Patterns of Perceived Control That Buffer Against Cognitive Decline in Midlife and Old Age

**DOI:** 10.1093/geronb/gbaf081

**Published:** 2025-05-21

**Authors:** Jeremy M Hamm, Jennifer R Turner, Margie E Lachman, Laura M Klepacz, Matthew J Pierce, Kelly Parker

**Affiliations:** Department of Psychology, North Dakota State University, Fargo, North Dakota, USA; Department of Psychology, University of Hawaii at Hilo, Hilo, Hawaii, USA; Department of Psychology, Brandeis University, Waltham, Massachusetts, USA; Department of Psychology, North Dakota State University, Fargo, North Dakota, USA; Department of Psychology, North Dakota State University, Fargo, North Dakota, USA; Department of Psychology, North Dakota State University, Fargo, North Dakota, USA; (Psychological Sciences Section)

**Keywords:** Cognitive aging, Control belief profiles, Domain-specific, Latent profile analysis, Person-centered approach

## Abstract

**Objectives:**

The relationship between domain-general or global perceptions of control and cognition is well-established. However, little is known about how these domain-general beliefs combine with domain-specific perceptions in central life domains to form multifaceted patterns of control that may buffer against cognitive declines in midlife and old age.

**Methods:**

We used 9-year data from the Midlife in the United States Study (*n* = 2,734, *M*_age_ = 55 years, range = 33–83; 58% female) to identify profiles of domain-general (personal mastery, perceived constraints) and domain-specific control over central life domains (health, work, finances, others’ welfare, child relationships, and partner relationship). We subsequently assessed profile differences in 9-year trajectories of cognitive aging and whether these differences became pronounced in old age.

**Results:**

Factor mixture models showed that 4 common profiles emerged: low control, family control, work control, and domain-specific control. Autoregressive ANCOVAs showed the family control and work control profiles experienced the least 9-year decline in executive functioning (*F*_3,2330_ = 3.46, *p* = .016). Moderation models showed the family control profile experienced less decline in executive functioning than the work control profile, but only in old age (*b* = −0.006, *p* = .020). Supplemental analyses showed profile differences in cognitive aging were (a) mediated by theory-derived process variables (positive and negative affect) and (b) extended to a broader suite of health-related developmental outcomes (functional limitations, chronic conditions, and mortality).

**Discussion:**

Findings inform lifespan theories of development by documenting meaningful patterns of domain-general and domain-specific control that have implications for healthy cognitive aging.

The beliefs people hold about their capacity to influence or control important outcomes in their lives matter. These perceptions reflect a core psychological resource that regulates motivation, emotion, and behavior and thereby buffers against age-related chronic diseases, functional losses, and cognitive decline (Hamm, Lachman, et al., 2025; Hong et al., 2021; Infurna et al., 2011; Lachman, 2006; Menec & Chipperfield, 1997; Windsor & Anstey, 2008). For example, Infurna and Gerstorf (2013) found that higher levels of perceived control predicted less episodic memory decline in a national U.S. sample of over 4,000 middle-aged and older adults. However, perceived control is a construct that consists not only of domain-general or global beliefs but also of domain-specific beliefs about control over central life domains (Lachman & Firth, 2004). Little is known about how these beliefs combine to form multifaceted profiles of control that may slow or accelerate rates of cognitive decline in midlife and old age. The present study thus used data from the national Midlife in the United States Study (MIDUS) to (a) identify common patterns of domain-general and domain-specific control in midlife and old age, (b) document profile differences in 9-year trajectories of cognitive functioning, and (c) evaluate whether profile differences became pronounced in old age.

Lachman’s process model of control provided a theoretical basis for our study (Lachman & Firth, 2004; Robinson & Lachman, 2016). This model specifies the motivation, affective, and health behavior pathways via which perceived control should buffer against declines in health and cognition. Particularly relevant to the present study, the process model addresses the multidimensional and multidomain nature of perceived control. Perceived control is posited to be multidimensional such that it consists of facets involving personal mastery and perceived constraints (Lachman, 2006; Lachman & Weaver, 1998b): Personal mastery refers to beliefs about one’s ability to perform specific actions to achieve goals, whereas perceived constraints refers to beliefs about external obstacles that undermine the efficacy of personal actions to achieve goals. Perceived control is posited to be multidomain such that it is not constant but rather can vary across central life domains that include health, work, and personal relationships, among others (Drewelies et al., 2019; Lachman & Firth, 2004; Lachman & Weaver, 1998a).

Domain-general control involves the global beliefs people hold about their influence over life in general. Previous research suggests that the different dimensions of domain-general control (mastery, constraints) are only moderately correlated (Hamm et al., 2023; Infurna & Mayer, 2015; Lachman & Weaver, 1998) and that their consequences may differ for developmental outcomes across adulthood, including cognitive aging. For example, recent work showed that rates of decline in episodic memory and executive functioning were reduced by approximately 22% over nearly a decade for older U.S. adults who reported declines in constraints (Hamm, Lachman, et al., 2025). In contrast, increases in mastery were unrelated to cognitive aging in this study. This pattern is consistent with related research that found constraints (vs. mastery) were a stronger predictor of subjective memory complaints, executive functioning, episodic memory, and cognitive impairment (Hong et al., 2021; Infurna et al., 2018; Khoo & Yang, 2020; Lee, 2016; Wong & Yang, 2023). These findings suggest that perceived control varies across dimensions and that constraints may be more tightly linked to cognitive aging. However, open questions remain about how different combinations of mastery and constraints may buffer against cognitive declines, especially when paired with varying levels of domain-specific control.

Domain-specific control involves the beliefs people hold about their influence over individual life domains such as their health, work, and personal relationships. Although less is known about the association between domain-specific control and cognitive aging, initial research indicates that control over central life domains may also play a protective role. Emerging evidence suggests that control over certain domains could potentially be more influential than others. For instance, research by Robinson and Lachman (2020) found that control over health and social interactions exhibited the strongest associations with episodic memory and executive functioning. These findings are consistent with other studies that point to control over health, over its subdomains (cognitive health and health behaviors), and over personal relationships as being more tightly coupled with cognition relative to control over other life domains such as work (Cerino et al., 2023; Lachman & Andreoletti, 2006; Lachman et al., 2009; Raldiris et al., 2021; Valentijn et al., 2006).

Taken together, this body of research points to the complex role of different dimensions and domains of control in healthy cognitive aging. However, critical open questions remain because past work has largely neglected the ecological reality that domain-general and domain-specific perceptions of control are likely to combine to buffer against cognitive declines (Lachman et al., 2009). Specifically, previous research has relied on variable-centered approaches such as regression that treat complementary control beliefs as competing predictor variables. This contrasts with person-centered methods, such as latent profile analysis (LPA), that enable the identification of multidimensional combinations of control beliefs that operate together in tandem. No studies to date have directly examined combinations or patterns of control across dimensions and domains. Some indirect support for such patterns comes from research examining control diversity (Drewelies et al., 2019) that focused on variability in stressor control across multiple life domains. Findings showed that individuals differed in their patterns of control, such that some had substantial variability across domains (e.g., high control over relational stressors but low control over work stressors), whereas others exhibited limited variability (e.g., low or high control over stressors across all domains). However, their analytic approach involved calculating a single entropy value that reduced combinations of control to the degree of variability across stressor domains, and Drewelies et al. (2019) did not consider the implications of entropy for cognitive functioning. Research using mixture modeling approaches such as LPA is needed to better capture how individuals differ in their multifaceted patterns of control across dimensions and life domains, and how such profiles may diverge in rates of cognitive aging.

Little is likewise known about which combinations of perceived control may be most protective against cognitive declines in later life. Past research that has focused on overarching, domain-general control beliefs provides indirect, albeit mixed, evidence that age may play a moderating role. Two early studies did not find support for age-moderated associations between perceived control and cognitive aging (Agrigoroaei & Lachman, 2011; Infurna & Gerstorf, 2013). However, subsequent research has observed stronger associations between perceived control and healthy cognitive functioning among older, rather than younger, adults (Oumohand et al., 2020; Raldiris et al., 2021; Windsor & Anstey, 2008). Longitudinal research using prospective designs is needed to systematically examine how multifaceted combinations of domain-general and domain-specific control may buffer against cognitive declines to a greater extent in old age.

The present study used 9-year data from the national MIDUS study to address our research objectives. The first objective was to identify common patterns of domain-general and domain-specific control in midlife and old age. Based on Lachman’s process model of control (Lachman & Firth, 2004; Robinson & Lachman, 2016), we focused on core indicators of both domain-general control (personal mastery, perceived constraints) and domain-specific control over central life domains (health, work, finances, others’ well-being, children, spouse or romantic partner). We thus adopted a person-centered analytic approach that enabled the identification of common profiles or patterns of control across all eight indicators of control. This more nuanced approach may better reflect the ecological reality that people simultaneously hold multiple control beliefs that encompass both broad (domain-general) and narrow facets (domain-specific; Lachman et al., 2009). We expected several distinct profiles to emerge that would broadly differ in the extent to which strong domain-general control beliefs were paired with high control over domains that selectively emphasized health, work/finances, or social/family relationships.

The second objective was to document profile differences in 9-year trajectories of cognitive aging. We focused on longitudinal changes in central indicators of cognitive functioning sensitive to early age-related declines: episodic memory and executive functioning (Hughes et al., 2018). We expected profiles that paired high domain-general control with high control over specific domains most implicated in cognitive aging, such as personal relationships and health (Cerino et al., 2023; Lachman et al., 2009; Robinson & Lachman, 2020), to buffer against cognitive declines relative to profiles characterized by low control across all indicators. The third objective was to evaluate whether profile differences in 9-year cognitive aging differed across midlife and old age. Based on theories of lifespan motivation and development (Carstensen, 2006; Carstensen et al., 1999; Heckhausen et al., 2010), we expected that the benefits of profiles characterized by higher levels of control over personal relationships in the form of close family ties would become pronounced in old age.

We also sought to extend prior research in two supplemental objectives. The first was to test whether profile differences in cognitive aging were mediated by theory-derived process variables involving positive and negative affect (Lachman, 2006; Robinson & Lachman, 2016). We focused on longitudinal changes in affect as mediating mechanisms because they reflect potentially important yet understudied pathways relative to other proposed mediators such as health behaviors, which have received more attention (Hamm, Lachman, et al., 2025; Infurna & Gerstorf, 2013; Robinson & Lachman, 2020). Specifically, we examined positive and negative affect as mediators based on Lachman’s process model (2006) and past research that has consistently linked perceived control to affect and affect to cognition (Castro-Schilo et al., 2019; Gerstorf et al., 2018; Hamm, Shane, et al., 2023; Hittner et al., 2020), but has yet to test affect as a theory-based pathway linking control to cognitive aging. The second supplemental objective was to examine the broader consequences of profile differences for healthy aging. We thus explored whether profile differences extended to longitudinal changes in other key health-related, developmental outcomes involving functional limitations, chronic conditions, and mortality (Heckhausen et al., 2010, 2013). These health outcomes have been consistently linked to domain-general control, but have yet to be considered in relation to multifaceted patterns of control across dimensions and life domains (Chipperfield et al., 2018; Hong et al., 2021; Infurna et al., 2011; Menec & Chipperfield, 1997).

## Method

### Participants and Procedures

We examined our research questions using data from the MIDUS National Longitudinal Study of Health and Well-being (Brim et al., 2004; Ryff et al., 2017). MIDUS is an ongoing national study of U.S. adults who were 25–75 years old at baseline assessment (1995–2013). Baseline data were assessed in 1995 (Wave 1; *n* = 7,108), and all willing participants were reassessed in 2004 (Wave 2; *n *= 4,963) and 2013 (Wave 3; *n *= 3,294). At Wave 2, an oversample of 592 African Americans residing in Milwaukee, Wisconsin was recruited into the MIDUS study. The current study focused on participants from Waves 2 and 3 because (a) data were not collected for the Milwaukee oversample at Wave 1 and (b) cognitive functioning was not assessed for any sample at Wave 1.

Inclusion criteria for the present study were that participants provided data at Wave 2 on our indicator variables for the latent profiles (domain-specific and domain-general facets of perceived control) and at Waves 2 and 3 for a least one of our primary outcome measures (episodic memory, executive functioning). These criteria allowed us to examine how profiles of perceived control predicted 9-year changes in cognitive functioning. At Wave 2, the analyzed sample (*n* = 2,734) had a mean age of 55±11 years (range = 33–83), was 58% female and 88% White, had an average household income of $73,262, and 70% had some postsecondary education. As is typical in longitudinal studies (Lindenberger et al., 2001; Radler & Ryff, 2010), participants in the current analyzed sample (who provided longitudinal data at Waves 2 and 3) were: more likely to be younger, female, have higher education and income, have fewer functional limitations, to report fewer perceived constraints and higher domain-specific control (over their health, finances, and others’ welfare), and to have higher episodic memory and executive functioning (*p*s < .001). The magnitudes of these differences were small (*d*s = 0.14–0.40; Cohen, 1988). Participants in the analyzed sample did not differ from those lost to attrition on personal mastery, work control, child control, spouse control, or race (*p*s > .075). MIDUS data collection was reviewed and approved by the Education and Social/Behavioral Sciences and the Health Sciences Institutional Review Boards at the University of Wisconsin-Madison.

### Study Measures

#### Facets of domain-general perceived control

The 12-item Sense of Control Scale was assessed at Wave 2 and captured two domain-general aspects of control involving personal mastery and perceived constraints (Hamm, Barlow, et al., 2023; Lachman & Weaver, 1998b). Participants indicated their agreement with the four mastery and eight constraint items using a 7-point scale (1 = *strongly agree*, 7 = *strongly disagree*). Mastery and constraint scores were derived by calculating mean scores of the reverse-coded items for each subscale, such that higher scores reflected higher levels of mastery (*α* = 0.73) and constraints (*α*s = 0.85). See Table 1 and Supplementary Table 1 in Supplementary Material for a summary of the sample characteristics and interitem correlations between the study variables.

**Table 1.** Sample Characteristics and Interitem Correlations

Variable	1	2	3	4	5	6	7	8	9	10	11	12	13	14	15	16	17	18
1. Age^b^	–																	
2. Sex (female)^a^	−0.00	–																
3. Race (minority)^a^	−0.08	0.07	–															
4. Education^b^	−0.11	−0.13	−0.13	–														
5. Income^b^	−0.22	−0.14	−0.16	0.37	–													
6. Health status^b^	−0.06	−0.04	−0.17	0.25	0.20	–												
7. Mastery^b^	−0.10	−0.04	0.03	0.05	0.11	0.16	–											
8. Constraints^b^	0.06	−0.06	0.04	−0.21	−0.21	−0.27	−0.44	–										
9. Health control^b^	−0.02	0.01	−0.01	0.07	0.07	0.43	0.23	−0.30	–									
10. Work control^b^	0.21	0.01	−0.06	0.09	0.08	0.20	0.14	−0.25	0.25	–								
11. Finances control^b^	0.10	−0.06	−0.04	0.08	0.15	0.20	0.20	−0.26	0.31	0.39	–							
12. Others control^b^	0.03	0.02	−0.00	0.13	0.05	0.19	0.14	−0.22	0.20	0.25	0.28	–						
13. Children control^b^	−0.19	0.02	0.10	−0.01	0.03	0.06	0.14	−0.14	0.12	0.06	0.07	0.13	–					
14. Spouse control^b^	0.05	−0.03	−0.02	−0.05	0.02	0.09	0.16	−0.19	0.18	0.15	0.26	0.18	0.22	–				
15. EM^b^	−0.24	0.22	−0.12	0.20	0.14	0.14	0.06	−0.14	0.09	0.01	0.01	0.05	0.04	−0.02	–			
16. EF^b^	−0.34	−0.14	−0.27	0.44	0.31	0.28	0.07	−0.19	0.10	0.04	0.04	0.08	0.03	−0.02	0.38	–		
17. ΔEM^bc^	−0.29	0.14	−0.02	0.08	0.12	0.08	0.07	−0.12	0.05	−0.01	0.00	0.04	0.06	0.00	0.00	0.18	–	
18. ΔEF^bc^	−0.28	−0.02	−0.03	0.06	0.12	0.06	0.06	−0.09	0.06	−0.07	−0.02	0.00	0.07	−0.02	0.05	0.01	0.23	–
* M*	54.87	1.58	0.12	7.45	73,262	3.65	5.60	2.63	7.79	7.40	7.06	7.62	7.62	7.91	0.08	0.07	−0.00	0.00
* SD*	11.31	0.49	0.33	2.53	60,016	0.96	1.06	1.21	1.76	2.56	2.39	2.24	2.24	2.03	0.93	0.67	0.84	0.47

*Notes*: Constraints = perceived constraints; EF = executive functioning; EM = episodic memory; Mastery = personal mastery; Others control = perceived control over others’ welfare; *SD* = standard deviation; Δ = regressed change; *n* range = 1,834–2,734. All correlations ≥ |.05| are significant at *p* < .05.

^a^Wave 1.

^b^Wave 2.

^c^Wave 3.

#### Facets of domain-specific perceived control

Perceived control was also assessed in six central life domains, including health, work, finances, others’ welfare, child relationships, and spouse or partner relationship. Consistent with previous research (Hamm et al., 2019; Lachman & Weaver, 1998a), perceived control in each domain was measured using the following single item: “How would you rate the amount of control you have over [relevant domain] these days?” Participants rated their perceived control on an 11-point scale (0 = *no control at all*, 10 = *very much control*).

#### Cognitive function

The Brief Test of Adult Cognition by Telephone (BTACT) was used to assess episodic memory and executive functioning at Waves 2 and 3 (Lachman & Tun, 2008; Tun & Lachman, 2006). Previous research with middle-aged and older adults has shown the BTACT to be a reliable and valid measure of central dimensions of cognition involving episodic memory and executive functioning (Hamm et al., 2020; Lachman et al., 2014; Tun & Lachman, 2006). Episodic memory was assessed using immediate and delayed recall tasks (free recall of 15 words). Executive functioning was assessed using measures of inductive reasoning, category verbal fluency, working memory span, processing speed, and attention switching and inhibitory control. See the Supplementary Material for further details on the BTACT.

Measures of episodic memory (based on two tests) and executive functioning (based on five tests) were calculated by averaging the *z* standardized values of their respective subtests at both waves (Hughes et al., 2018). Higher scores reflect better average episodic memory and executive functioning performance. Consistent with previous research, we used the raw Wave 2 means and standard deviations to generate the *z* scores for each test at Wave 3. We generated our primary outcome measures of regressed (residualized) change in episodic memory and executive function by regressing Wave 3 scores on the corresponding baseline (Wave 2) levels of each measure (Cohen et al., 2013; Maxwell et al., 2017; Tennant et al., 2022). Residuals from these analyses were saved and used as indicators of regressed, longitudinal change in episodic memory and executive functioning (Cohen et al., 2013). Scores of 0 on our regressed change measures roughly reflect average (expected) sample rates of 9-year decline in episodic memory (raw decline *M* = −0.115) and executive functioning (raw decline *M *= −0.256). Positive values indicate less decline than expected in this sample, and negative values indicate steeper (more) decline than expected.

#### Demographic covariates

Age, sex, race, education, income, and self-reported physical health are well-established correlates of perceived control and cognitive functioning and were thus included as covariates in the main analyses (Dixon & Lachman, 2019; Hamm, Parker, et al., 2024; Hughes et al., 2018; Lachman et al., 2014; Robinson & Lachman, 2018; Tran et al., 2014). Age in years was assessed at Wave 2. Sex (1 = *male*, 2 = *female*) and race (0 = *White*, 1 = *non-White*) were assessed at Wave 1. Level of formal education completed (1 = *no school or grade school*, 12 = *doctoral degree*) and total household income in U.S. dollars were assessed at Wave 2. Physical health status was rated using a 5-point scale (1 = *excellent*, 5 = *poor*). Scores were reverse-coded so that higher scores reflected better physical health.

### Rationale for Analyses

Analyses were conducted in a stepwise fashion. Step 1 involved person-centered, factor mixture models (FMMs) with heterogeneous variances to identify subgroups of individuals with similar patterns of domain-general (mastery, constraints) and domain-specific perceived control (overwork, child relationships, spouse or partner relationship, health, finances, and others’ welfare) at Wave 2. FMMs reflect an extension of traditional LPAs that relaxes the stringent and often-unrealistic assumption of conditional independence (i.e., that there is no covariation between the indicator variables within each profile; Morin & Marsh, 2015). See Supplementary Material for a more detailed overview of FMM. FMM models were assessed with Mplus 8 using maximum likelihood robust estimation and the MplusAutomation package (Muthén & Muthén, 1998–2017; Hallquist & Wiley, 2018). Missing data were handled using FIML so that participants who provided data on at least one indicator variable were included in the analyses.

Step 2 involved autoregressive analyses of covariance (ANCOVA) procedures to test for profile differences in subsequent 9-year changes in two central indicators of cognitive functioning (episodic memory, executive functioning). Step 3 involved autoregressive OLS regression analyses that incorporated age as a moderator variable to assess whether profile differences in trajectories of cognitive functioning became pronounced in old age. Step 2 models, Step 3 models, and all supplemental analyses controlled for age, sex, race, education, income, and self-reported physical health, and baseline levels of each outcome measure (i.e., autoregressive effects).

## Results

### Step 1: Latent Profiles of Perceived Control

**Figure 1.**
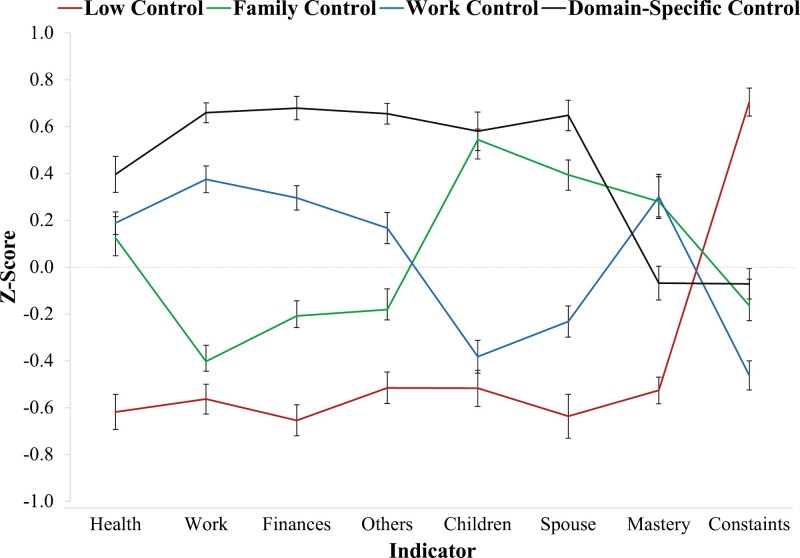
Results from the *k* = 4 profile model of domain-specific and domain-general perceived control. The low control profile (*n* = 670) reflected individuals with very low levels of control across all aspects of domain-specific and domain-general control. The family control profile (*n* = 663) reflected individuals with high levels of control over their family relationships with children and spouses and relatively average levels of control over most other aspects (with above-average levels of mastery). The work control profile (*n* = 793) had higher levels of control over work and finances, lower levels of control over family relationships with children and spouses, and above-average levels of mastery and below-average levels of control. The high domain-specific control profile (*n* = 608) had high levels of control over all aspects of domain-specific control but had average levels of mastery and constraints.

Results for the FMMs are shown in Table 2 and indicate the four-profile solution produced the best model fit based on multiple criteria. See Supplementary Material for further details on model selection criteria and model fit comparisons. The latent profiles that emerged in the four-profile solution are depicted in Figure 1 and were labeled low control (*n* = 670; 25%), family control (*n* = 663; 24%), work control (*n* = 793; 29%), and domain-specific control (*n* = 608; 22%). As shown by the profile means in Figure 1, profiles differed substantially in their levels of domain-specific and domain-general control. The low control profile had very low levels of control across all aspects of domain-specific and domain-general control. The family control profile had high levels of control over their family relationships with children and spouses, below-average levels of control over work and finances, relatively average levels of control over other life domains, and above-average levels of domain-general control (higher mastery and slightly lower constraints). The work control profile had higher levels of control over work and finances, lower levels of control over family relationships with children and spouses, and above-average levels of domain-general control (higher mastery and lower constraints). The high domain-specific control profile had high levels of control over all aspects of domain-specific control but had average levels of domain-general mastery and constraints.

**Table 2.** Model Fit for Latent Profiles of Domain-Specific and Domain-General Control (*k* = 2–6 Profile Solutions)

No. of profiles	LL	Free par.	AIC	BIC	SABIC	BLRT *p*	LMR *p*	Entropy	Profile size		
									< 1%	< 5%	< 10%
2	−39,035	41	78,151	78,394	78,263	<.001^a^	<.001	.700	0	0	0
3	−38,522	58	77,161	77,504	77,320	<.001	<.001	.669	0	0	0
**4**	−**38,187**	**75**	**76,524**	**76,968**	**76,729**	**<.001** ^*^	**<.001**	**.650**	**0**	**0**	**0**
5	−37,961	92	76,105	76,649	76,357	<.001^*^	.222	.685	0	0	1
6	−37,798	109	75,814	76,459	76,112	<.001^*^	.630	.697	0	0	2
Interpretation	*Lower* values better		*Lower* values better	*Lower* values better	*Lower* values better	*Significant* values support tested model over model with one less profile	*Significant* values support tested model over model with one less profile	*Higher* values better	*Fewer* profile sizes with < 1%, < 5%, < 10% better		

*Notes*: AIC = Aikake information criterion; BIC = Bayesian information criterion; BLRT = bootstrapped likelihood ratio test; Free par. = number of free parameters; LL = loglikelihood; LMR = Lo–Mendell–Rubin adjusted likelihood ratio test; SABIC = sample-size adjusted BIC. Profile size refers to number of latent profiles that contain < 1%, < 5%, or < 10% of the sample. Bold font indicates the best fitting model selected. All models allowed for heterogeneous variances across classes in the indicator variables.

^a^ *p* Value may not be trustworthy due to local maxima.

We examined profile differences in baseline age, sex, race, education, income, and cognitive functioning (see Supplementary Material). Briefly, the family control profile was younger than the other profiles, whereas the work control profile had fewer women, more White individuals, and higher education and income. The work control and family control had higher initial episodic memory and executive functioning. Age, sex, race, education, income, and cognitive functioning were controlled for in all subsequent analyses to ensure profile effects on the outcome variables were not due to baseline differences in these variables. We also conducted FMM sensitivity analyses based on the full sample that had cross-sectional data at Wave 2 (*n* = 4,795). Findings were consistent with our main analyses of the longitudinal sample (*n* = 2,734) such that the four-profile model exhibited the best fit, and the same four profiles emerged (see Supplementary Material).

### Step 2: Profile Differences in 9-Year Cognitive Functioning

Separate analyses of covariance (ANCOVAs) tested whether control profiles differed in 9-year regressed change in episodic memory and executive functioning. ANCOVAs controlled for age, sex, race, education, income, self-reported physical health, and baseline levels of each outcome measure (i.e., autoregressive effects). Controlling autoregressive effects permitted an examination of control profile differences in longitudinal, regressed changes in the cognitive functioning outcome measures, such that variance due to baseline levels of the outcome measures was statistically partialed out (Cohen et al., 2013; Maxwell et al., 2017; Tennant et al., 2022).

Separate ANCOVAs indicated there was an omnibus control profile effect for executive functioning, *F*_3,2330_ = 3.46, *p* = .016, but not for episodic memory, *F*_3,2585_ = 1.08, *p* = .356 (see Supplementary Table 3). Covariate-adjusted, pairwise comparisons showed that the family control profile experienced less decline in 9-year executive functioning relative to the low control profile (*M*_diff_ = 0.06, *SE* = 0.027, *t*_2330_ = 2.22, *p* = .027, Cohen’s *d* = 0.08) and the domain-specific control profile (*M*_diff_ = 0.07, *SE* = 0.028, *t*_2330_ = 2.42, *p* = .016, Cohen’s *d* = 0.09). Similarly, the work control profile also experienced less decline in 9-year executive functioning relative to the low control profile (*M*_diff_ = 0.05, *SE* = 0.027, *t*_2330_ = 2.04, *p* = .041, Cohen’s *d* = 0.07) and the domain-specific control profile (*M*_diff_ = 0.06, *SE* = 0.026, *t*_2330_ = 2.36, *p* = .019, Cohen’s *d* = 0.08).

**Figure 2.**
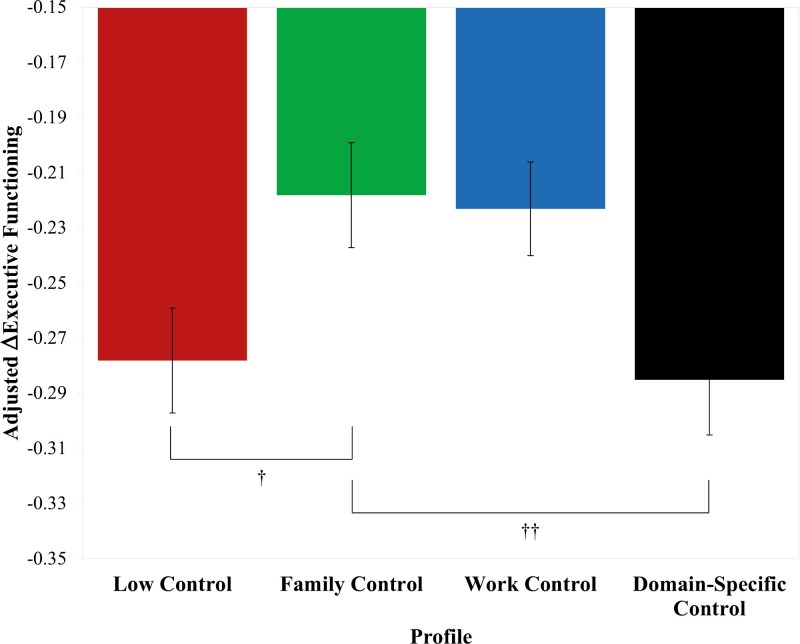
Contextualized effect sizes of profile differences on regressed change in executive functioning. Estimates suggested rates of 9-year decline in executive functioning were respectively reduced by approximately 22% and 24% for the family control profile versus the low control and domain-specific control profiles. Predicted values were adjusted for model covariates and for (raw) average sample declines of −0.256 units in executive functioning. ^†^22% reduction in rate of decline. ^††^24% reduction in rate of decline.

To contextualize the practical significance and effect sizes of the profile differences, we generated predicted values (PVs) that adjusted for raw, average sample declines of −0.256 units in executive functioning over the 9-year follow-up. Small but meaningful differences emerged in predicted executive functioning scores for those in the family control profile (−0.218) relative to those in the low control (−0.278) and domain-specific control profiles (−0.285). These estimates suggest that rates of 9-year decline in executive functioning were reduced by nearly 25% (−0.218 vs. −0.278, −0.285) for individuals in the family control profile (see Figure 2). Similar benefits were observed for those in the work control profile.

### Step 3: Moderated Profile Differences in 9-Year Cognitive Functioning

**Figure 3.**
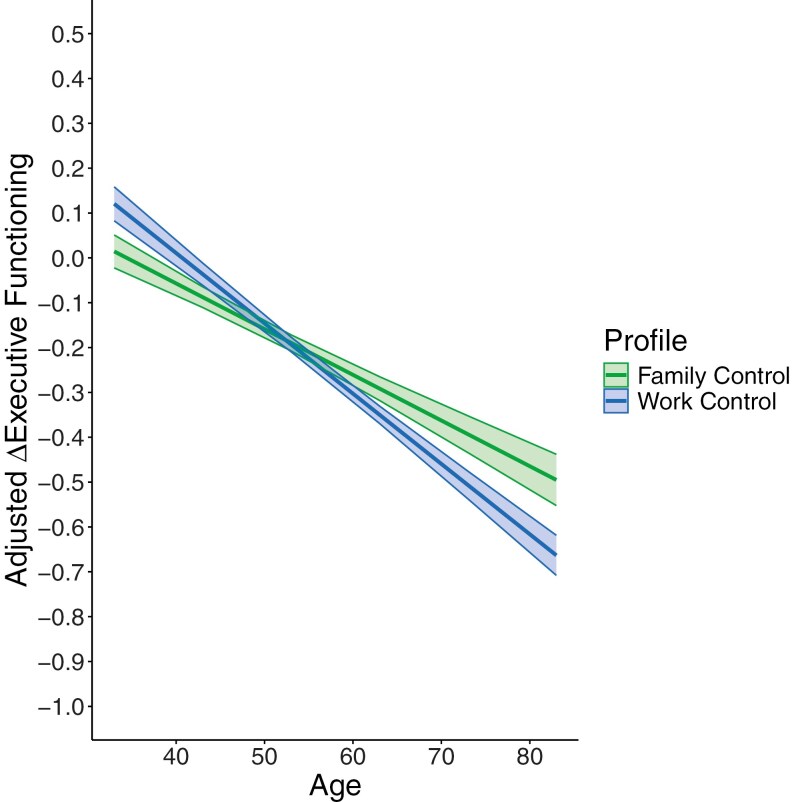
Age × Work Control interaction predicting 9-year regressed change in executive functioning. Predicted values were adjusted for model covariates and for (raw) average sample declines of −0.256 units in executive functioning.

Autoregressive OLS regression models tested whether profile differences were moderated by age. Analyses were conducted with dummy-coded profile variables that reflected low control, work control, and domain-specific control (reference group = family control). Age was treated as a continuous moderator variable. Results showed that age moderated profile differences for executive functioning, but not for episodic memory. Specifically, an Age × Work Control interaction emerged (*b* = −0.006, *SE* = 0.002, *p* = .020) and indicated that executive functioning differences between those in the work control and family control profiles became stronger in old age (see Figure 3). We probed the interaction using Johnson–Neyman spotlight approach (Hayes, 2017). Results showed that the work control (vs. family control) profile experienced significantly greater declines in executive functioning among older adults aged 68–83 years (*p*s < .05): from *b* = −0.09, *SE* = 0.044, *p* = .048 at age 68 to *b* = −0.17, *SE* = 0.075, *p* = .025 at age 83. There were no significant differences between the profiles from ages 33 to 67.

### Supplemental Analyses

#### Mediation analyses

Supplemental, autoregressive mediation models tested whether 9-year regressed changes in theory-derived process variables (positive and negative affect) mediated links between the dummy-coded control profiles and cognitive functioning trajectories. See Supplementary Material for details on each measure of affect and bootstrapped procedures that tested mediation. Results of autoregressive, OLS regression models showed that individuals in the family control and work control (vs. low control) profiles experienced greater longitudinal increases in positive affect and declines in negative affect, which in turn predicted less decline in 9-year episodic memory and executive functioning (see Supplementary Tables 4 and 5). Bootstrapped tests of indirect effects showed that the family control and work control (vs. low control) profiles experienced slower episodic memory and executive functioning declines due in part to the mediating influence of positive and negative affect (see Supplementary Table 6).

#### Health-related developmental outcomes analyses

Supplemental analyses tested whether profile differences emerged for a broader suite of health-related developmental outcomes including functional status, chronic conditions, and mortality. See Supplementary Material for details on each supplemental outcome measure. Separate, autoregressive ANCOVAs indicated there were omnibus control profile effects for both longitudinal changes in 9-year functional limitations (*F*_3,2368_ = 6.72, *p* < .001) and chronic conditions (*F*_3,2350_ = 4.92, *p* = .002). Pairwise comparisons showed that functional limitations and chronic conditions increased at a slower rate for individuals in the family control, work control, and domain-specific control profiles relative to the low control profile (see Supplementary Table 7).

A Cox proportional hazard regression model using dummy-coded profile variables showed that only the low control profile had a marginally increased risk of death relative to the family control profile (HR = 1.32, *CI*s = 0.991–1.763, *p* = .057; see Supplementary Table 8). Specifically, those in the low control (vs. family control) profile had a 32% increase in risk of death. Mortality findings were consistent in sensitivity analyses that evaluated the robustness of the Cox regression models using the full sample that had cross-sectional data at Wave 2 and who died following the second interview between 2004 and 2022 (vs. following the third interview between 2013 and 2022). Those in the low control profile were at increased risk of death relative to the family control (HR = 1.23, *CI*s = 1.046–1.449, *p* = .013) and the work control profiles (HR = 1.31, *CI*s = 1.118–1.542, *p* < .001; see Supplementary Table 9).

## Discussion

Our study sheds new light on how different dimensions and domains of perceived control combine to buffer against cognitive declines in midlife and old age. Findings inform lifespan theories of control by identifying meaningful patterns (profiles) of domain-general and domain-specific control that commonly occur in midlife and old age. Results also advance the literature in documenting how certain profiles that emphasize family control, while maintaining above-average levels of domain-general control, were at reduced risk of cognitive declines, and that this advantage became pronounced in old age. Supplemental findings indicated that profile differences in cognitive aging were mediated by theory-derived process variables (positive and negative affect) and extended to a broader suite of core developmental outcomes in later life (functional limitations, chronic conditions, and mortality).

Our study is the first to adopt a person-centered approach to identify commonly occurring profiles of perceived control across dimensions and domains. In contrast to variable-centered methods (Hamm, Lachman, et al., 2025; Hong et al., 2021; Infurna et al., 2018; Robinson & Lachman, 2020), this approach may better reflect the ecological reality that people simultaneously hold an array of beliefs about their control over life in general and over specific areas of their lives. Our findings suggest that these domain-general and domain-specific beliefs operate in tandem and can be captured by complex rather than main effect combinations. Consistent with multidimensional and multidomain models of control (Lachman & Firth, 2004; Robinson & Lachman, 2016), the present results thus contribute to a more nuanced understanding of perceived control in providing initial evidence that domain-general and domain-specific control beliefs coexist in meaningful mixtures rather than in isolation.

The four profiles that emerged exhibited noteworthy differences in their combinations of control. Patterns evident in the family control and work control profiles offer an interesting contrast in domain-specific emphases, with each notably paired with above-average levels of domain-general control. Middle-aged and older adults in the family control profile reported high control over their family relationships (children and spouses), but relatively low control over their work-related achievements (work and finances). This pattern slightly differs from prior research that had found that, on average, control over child relationships tended to decline as people age (Lachman et al., 2009; Shane & Heckhausen, 2016). However, these studies also observed substantial variability in rates of average change, which suggests that many individuals may retain high levels of control over their child relationships as they age. In contrast to the family control profile, those in the work control profile reported relatively high control over work-related achievements, but much less control over family relationships.

These diverging emphases in the family versus work control profiles are broadly consistent with propositions stemming from theories of lifespan development and motivation (Carstensen et al., 1999; Carstensen & Hershfield, 2021; cf., Heckhausen et al., 2010). For example, the family control profile roughly captures a pattern in line with prioritizing emotionally close partners, which socioemotional selectivity theory (SST) posits becomes an increasingly important goal as people age and become cognizant of limited time remaining in life (Carstensen, 2006). Conversely, the work control profile roughly captures a pattern consistent with prioritizing exploration, knowledge gains, and achievement, which SST posits is more heavily valued in early adulthood and midlife when people generally perceive more extended time horizons (Carstensen, 2006).

This difference in emphasis is particularly interesting in the context of our adult lifespan sample that included young, middle-aged, and older adults because SST has suggested midlife reflects a rough inflection point (Carstensen et al., 1999). In particular, goals regarding exploration and knowledge gains start to become less salient in midlife, whereas goals focused on emotionally close partners take on greater meaning (Carstensen & Hershfield, 2021). In this respect, it is somewhat surprising that the work control profile was slightly older (aged 56) than the family control profile (aged 50). This may point to a relatively fluid process of shifting goal prioritizations that are based on perceptions of time remaining rather than chronological age (which reflects a proxy for time remaining; Carstensen, 2006). However, maintaining an (off-time) emphasis on knowledge gains and achievement could eventually have detriments for those in the work control profile if goal reprioritization does not occur in old age.

Findings from our Step 2 analyses revealed that the control profiles differed in their rates of cognitive aging. Small but practically significant consequences emerged when contrasting the family and work control profiles to the low control and domain-specific control profiles. Contextualized effect sizes suggested that rates of 9-year decline in executive functioning were reduced by nearly 25% for middle-aged and older adults in the family and work control profiles. Noteworthy is that high levels of control across all domains did not appear to be as protective in the absence of strong perceptions of domain-general control. That is, individuals in the domain-specific control profile experienced rates of executive functioning decline that were steeper than their peers in the family and work control profiles and more in line with those in the low control profile. Such a pattern implies that high domain-specific control may need to be paired with at least moderately strong perceptions of domain-general control to protect against cognitive declines in midlife and old age. These findings are broadly in line with recent research that suggests certain domain-general dimensions of control may become more consequential for cognitive aging, but have yet to examine the role of multidimensional and multidomain patterns of control (Hamm, Lachman, et al., 2025; Hong et al., 2021).

We found that the cognitive benefits of family control became pronounced in later life. Although both the family control and work control profiles buffered against executive functioning declines compared to the other profiles, in old age (68+ years), family control had greater cognitive benefits. Restated, the work control profile experienced greater declines in executive functioning than the family control profile, but only in old age. Although this may be due in part to reduced relevance of work in later life (but see Hamm et al., 2019 for a discussion of work engagement in old age), our findings are consistent with SST and other lifespan motivation theories that emphasize the age-graded nature of developmental goal pursuit. These theories suggest it may become increasingly adaptive to prioritize and maintain control over close familial relationships in old age, rather than maintaining control over knowledge gains and achievement (Carstensen, 2006; Carstensen et al., 1999; Heckhausen et al., 2010). Our results are also in line with past research that has revealed the importance of preserving strong relationships and social engagement in late life to buffer against losses in cognitive functioning (Fingerman et al., 2020; Zahodne, 2021).

Findings from our supplemental analyses are among the first to show that theory-derived process variables involving positive and negative affect mediated the link between perceived control and cognitive aging. These results are consistent with past research that had documented links between perceived control, positive affect, and cognition (e.g., Gerstorf et al., 2018; Hittner et al., 2020) but had yet to systematically evaluate the control-affect-cognition sequence proposed by Lachman’s (2006) process model of control. Findings suggest that perceived control may influence cognitive aging indirectly via pathways that include longitudinal changes in trait-level affect. This extends prior work that had focused almost exclusively on health behavior pathways that underlie the benefits of perceived control (Hamm, Lachman, et al., 2025; Infurna & Gerstorf, 2013; Robinson & Lachman, 2018).

Our supplemental findings also point to the broader health consequences of domain-general and domain-specific combinations of control. Results showed the pattern of profile differences extended to other central developmental outcomes in midlife and old age that included chronic disease, functional limitations, and mortality risk (Heckhausen et al., 2013). Noteworthy was that all three profiles that maintained high control on at least one domain-general or domain-specific facet were buffered against steep declines in physical health (chronic disease, functional limitations). This implies, to some extent, that high control beliefs over life in general or over several key domains can compensate for lower control beliefs in other domains (Heckhausen et al., 2019; Krause, 2007; Lachman et al., 2009). However, this pattern did not extend to mortality risk, such that only those in the family control profile, and, to a lesser extent the work control profile, had a reduced risk of death over the longitudinal follow-up. These mortality findings are broadly in line with SST and point to the benefits of prioritizing control over close familial relationships in late life (Carstensen, 2006).

Although our study is supported by the use of multiple dimensions and domains of control and prospective 9-year data on cognitive functioning in a large national sample, it is not without limitations. First, despite the rich data on control available in MIDUS, this study did not assess dimensions of control (mastery and constraints) within each domain. That is, MIDUS collected data on these two core dimensions of control at the domain-general level but not at the domain-specific level. Further research is needed to examine the role of more nuanced combinations of domain-general and domain-specific perceptions of mastery and constraints. Second, our study did not consider the role of changes in profiles of control over time because our focus was on how individual differences in control profiles prospectively predicted cognitive aging over nearly a decade. Future research is needed to examine the consequences of profile changes for cognitive functioning. Second, although we included the Milwaukee oversample of Black participants, the MIDUS sample was largely White and upper-middle class. Further research is needed to replicate these findings in samples that are more racially and socioeconomically diverse.

In sum, the present findings provide evidence that domain-general and domain-specific control beliefs combine to form meaningful profiles that have consequences for healthy cognitive aging. Participants in our national sample who paired high levels of control over their family relationships with above-average control over their lives in general experienced the least decline in their executive functioning over a 9-year follow-up. Findings also suggest that the cognitive benefits of this family control profile became pronounced in old age. Results also have practical implications for the development of evidence-based interventions to buffer against cognitive declines and point to the potential value of targeting changes not only in perceptions of control over life in general but also in control over key domains that include close relationships.

## Supplementary Material

gbaf081_suppl_Supplementary_Tables_S1-S9

## Data Availability

MIDUS data and materials are available after registration from the Inter-University Consortium for Political and Social Research: https://www.icpsr.umich.edu/icpsrweb/ICPSR/series/203. Analyses were not preregistered.
