# Supplementary material for: Patterns of Perceived Control That Buffer Against Cognitive Decline in Midlife and Old Age
Source: J Gerontol B Psychol Sci Soc Sci. 2025 May 21;80(7):gbaf081. doi: 10.1093/geronb/gbaf081 (PMC12202204; doi:10.1093/geronb/gbaf081)
Supplement: gbaf081_suppl_Supplementary_Tables_S1-S9 [file gbaf081_suppl_supplementary_tables_s1-s9.docx]

***The Journals of Gerontology, Series B: Psychological Sciences and Social Sciences* Supplementary Material: Hamm et al. Patterns of Perceived Control that Buffer Against Cognitive Decline in Midlife and Old Age.**

**Method**

**Main Study Measures of Cognitive Functioning (BTACT)**

The Brief Test of Adult Cognition by Telephone (BTACT) assessed episodic memory and executive functioning at Waves 2 and 3 (Lachman & Tun, 2008; Tun & Lachman, 2006). Previous research with middle-aged and older adults has shown the BTACT to be a reliable and valid measure of central dimensions of cognition involving episodic memory and executive functioning (Lachman et al., 2014; Tun & Lachman, 2006). A detailed summary of the BTACT can be found elsewhere (Hughes et al., 2018; Lachman et al., 2010, 2014).

The BTACT battery includes two cognitive tests to assess episodic memory and five tests to evaluate executive functioning (Lachman et al., 2014). Episodic memory was assessed using immediate and delayed recall tasks (free recall of 15 words). Executive functioning was assessed using measures of inductive reasoning (completing patterns in a number series), category verbal fluency (number of animal names produced in one minute), working memory span (backward digit span), processing speed (number of digits produced counting backwards from 100 in 30 seconds), and attention switching and inhibitory control (Stop and Go Switch Task). The Stop and Go Switch Task comprised a reaction time test involving normal (respond GO to stimulus GREEN and STOP to stimulus RED) and reverse conditions (respond STOP to stimulus GREEN and GO to stimulus RED; Tun & Lachman, 2008). For the executive functioning measure, we used a recommended filter that retained data for only participants with valid scores on the Stop and Go Switch Task (Lachman et al., 2014; Tun & Lachman, 2008). Valid scores were those in which there were no technical malfunctions, the participant understood the task, and the participant was not distracted by external events.

**Supplemental Mediator Variables and Health-Related Developmental Outcomes**

**Positive and negative affect.** An abbreviated version of the Positive and Negative Affect Schedule (PANAS) was used to measure positive and negative affect at Waves 2 and 3 (Watson et al., 1988). Using a five-point scale (1 = *all of the time*, 5 = *none of the time*), participants were asked to report how frequently they experienced four positive (enthusiastic, attentive, proud, active) and five negative emotions (afraid, jittery, irritable, ashamed, upset) over the past 30 days. Scores were reverse-coded and averaged so that higher scores reflected higher levels of positive and negative affect. We generated our supplemental mediator measures of regressed (residualized) change in positive and negative affect by regressing Wave 3 scores on corresponding baseline levels of positive (*M* = 0.00, *SD* = 0.64, range = -3.38-2.24) and negative affect at Wave 2 (*M* = 0.00, *SD* = 0.46, range = -1.64-3.70).

**Functional limitations.** Activities of daily living (ADL limitations) were assessed at Waves 2 and 3. Participants reported the extent to which health limited their ability to perform 7 ADLs using a 4-point scale (1 = *a lot*, 4 = *not at all*; e.g., carrying groceries, climbing several flights of stairs, etc.). Scores were reverse coded so that higher scores reflected greater functional limitations. We generated our supplemental outcome measure of regressed (residualized) change in functional limitations by regressing Wave 3 scores on corresponding baseline levels at Wave 2 (*M* = 0.00, *SD* = 0.71, range = -2.59-2.55).

**Chronic conditions.** Chronic conditions were assessed at Waves 2 and 3 with a 30-item checklist that included diabetes, hypertension, stroke, and arthritis. Chronic conditions were indexed at each wave by summing the total number of conditions diagnosed or treated over the past 12 months (0 = *no*, 1 = *yes*). We generated our supplemental outcome measure of regressed (residualized) change in chronic conditions by regressing Wave 3 scores on corresponding baseline levels at Wave 2 (*M* =0.00, *SD* = 2.52, range = -23.14-16.23).

**Mortality.** Mortality status (0 = *alive*, 1 = *deceased*) and date of death was determined from National Death Index reports, tracing from mortality close out interviews, and sample maintenance. Survival times were calculated as the interval in months from the date of Wave 2 interview to the date of death (*M* = 172 months, range = 103-224 months). Survival times for participants still living were censored at 225 months. Seventeen percent (473/2734) of participants died between 2013 and 2022.

**Rationale for Factor Mixture Model Analyses (Step 1)**

We conduced person-centered, factor mixture models (FMMs) with heterogeneous variances to identify subgroups of individuals with similar patterns of domain-specific and -general perceived control at Wave 2. FMMs reflect an extension of traditional latent profile analyses (LPA) that relaxes the stringent and often-unrealistic assumption of conditional independence (i.e., that there is no covariation between the indicator variables within each profile; Morin & Marsh, 2015). FMMs account for covariance between the indicator variables by estimating a higher-order, continuous, latent factor that is based on the profile indicator variables and specified to be invariant across profiles (Perera & Ganguly, 2018).

FMM approaches also enable the estimation of latent profiles that better capture structural differences (shape effects) across different aspects of perceived control, which reflect the tendency for individuals to exhibit distinct patterns of vulnerability and strength in their combinations of control. This is achieved by incorporating the higher order, latent factor that captures level effects in a broad and overarching global sense of control, which reflect the tendency for some individuals to have low, medium, or high levels across all aspects of control (Morin & Marsh, 2015).

FMM models were assessed with Mplus 8 using maximum likelihood robust estimation and the MplusAutomation package (Muthén & Muthén, 1998-2015; Hallquist & Wiley, 2018). Missing data were handled using full information maximum likelihood so that participants who provided data on at least one indicator variable were included in the analyses. We estimated FMM models with varying numbers of profiles, ranging from two through six profiles (Marsh et al., 2009). As recommended by Infurna and colleagues (Infurna & Grimm, 2018; Infurna & Jayawickreme, 2019), we estimated heterogenous variance models which allowed the variances of all indicator variables to differ across profiles.

**Results**

**Step 1 Model Selection Criteria and Fit for the Latent Profile Analyses**

**Model selection criteria.** Model selection was guided by theory, interpretability, fit statistics, and profile size (Infurna & Grimm, 2018; Marsh et al., 2009; Orpinas et al., 2015; Pastor & Gagné, 2013). Several recommended fit indices were used (Nylund et al., 2007): the Aikake information criterion (AIC), the Bayesian information criterion (BIC), the sample-size adjusted BIC (SABIC), the bootstrapped likelihood ratio test (BLRT), and the Lo-Mendell-Rubin (LMR) adjusted likelihood ratio test. Lower values of AIC, BIC, and SABIC and significant BLRT and LMR tests indicate better fitting models. Although entropy values provided a measure of classification quality (range from 0-1 where higher values indicate clearer profile separation), they were not used to determine model selection based on recommendations by Lubke and Muthén (2007) and Morin and Marsh (2015). Optimal model solutions contain few profiles with less than 5% of the total sample and are parsimonious in adequately accounting for the complexity of the data with the fewest latent profiles (DiStefano & Kamphaus, 2006; Infurna & Grimm, 2018; Jung & Wickrama, 2008; Samuelsen & Raczynski, 2013).

**Model fit.** AIC, BIC, and SABIC values declined as number of profiles (model complexity) increased, and BLRT and LMR tests were all statistically significant until the 5-profile model. This was expected because our sample size was reasonably large and the present fit statistics are sample size dependent (see Marsh et al., 2009). Elbow plots were therefore generated to provide a graphical summary of the information criterion indices and assist in model selection (Petras & Masyn, 2010; Morin et al., 2011). These plots show the marginal gain in fit associated with increases in the number of profiles (increased model complexity). Results suggested the 4-profile model produced the largest marginal gain in fit (see Supplementary Figure 1 in Supplementary Material). Only the 2-, 3-, and 4-profile models contained no profiles with < 10% of the total sample. Balancing the findings, the 4-profile model was selected because it had: the largest marginal gain in fit across the AIC, BIC, and SABIC indices; significant BLRT and LMR test statistics; no profiles with < 10% of the sample; and clear interpretability.

**Step 1 Profile Differences in Demographic Characteristics**

Profile differences emerged for baseline demographic characteristics including age, sex, race, education, income, and cognitive functioning. The family control profile was younger than the other profiles (*M*s = 50 vs. 56-58). The work control profile had a lower proportion of women (49% vs. 56%-65%), a higher proportion of White individuals (92% vs. 85%-87%), and had higher levels of education (*M*s = 8.5 vs. 6.6-7.4) and income (*M*s = $95,231vs. $52,033-$72,557) than the other profiles. The work control and family control had higher initial episodic memory (*M*s = .20 to .21 vs. -.03 to -.12) and executive functioning (*M*s = .14 to .27 vs. -.04 to -.16) than the other profiles. We controlled for these sociodemographic characteristics in addition to physical health status in the subsequent analyses that tested for profile differences in cognitive functioning.

**Step 1 Sensitivity Analyses using the Cross-Sectional Sample at Wave 2**

Our main Step 1 models were based on participants with longitudinal data at Waves 2-3 because a primary aim was to test for profile differences in 9-year trajectories of cognitive functioning. Sensitivity analyses evaluated the robustness of the observed latent profiles using the full sample that had cross-sectional data at Wave 2 (*n* = 4,795). FMM results were consistent with the main analyses such that the 4-profile model exhibited the best fit, and the same four profiles emerged (see Supplementary Figure 2).

**Bootstrapped Approach for Supplemental Mediation Analyses**

We tested indirect effects (mediation) for significance using Hayes PROCESS macro and a bootstrap approach that employed 95% confidence intervals (Hayes, 2017; Preacher & Hayes, 2008). Mediation was confirmed if zero fell outside the confidence interval based on 5,000 samples of the unstandardized beta weights.

Supplementary Table 1

*Descriptive Characteristics of the Participants*

| Variable | *M* ± *SD* or *n* (%) | Range |
| --- | --- | --- |
| Age^b^ | 54.87 ± 11.31 | 33-83 |
| Sex^a^ |  |  |
| Male | 1152 (42.3%) |  |
| Female | 1573 (57.7%) |  |
| Race^a^ |  |  |
| White | 2336 (87.6%) |  |
| Black | 265 (9.9%) |  |
| Native American | 7 (0.3%) |  |
| Asian or Pacific Islander | 11 (0.4%) |  |
| Other | 31 (1.2%) |  |
| Multiracial | 17 (0.6%) |  |
| Education^b^ | 7.45 ± 2.53 | 1-12 |
| Income^b^ | 73262 ± 60016 | 0-300000 |
| Health status^b^ | 3.65 ± 0.96 | 1-5 |
| Mastery^b^ | 5.60 ± 1.06 | 1-7 |
| Constraints^b^ | 2.63 ± 1.21 | 1-7 |
| Health control^b^ | 7.79 ± 1.76 | 0-10 |
| Work control^b^ | 7.40 ± 2.56 | 0-10 |
| Finances control^b^ | 7.06 ± 2.39 | 0-10 |
| Others control^b^ | 7.62 ± 2.24 | 0-10 |
| Children control^b^ | 7.61 ± 2.40 | 0-10 |
| Spouse control^b^ | 7.91 ± 2.03 | 0-10 |
| Episodic memory^b^ | 0.08 ± 0.93 | -2.50-3.64 |
| Executive functioning^b^ | 0.07 ± 0.67 | -2.67-2.34 |
| Episodic memory^c^ | -0.03 ± 1.00 | -2.94-3.83 |
| Executive functioning^c^ | -0.18 ± 0.75 | -5.63-2.02 |

*Note.* Mastery = personal mastery. Constraints = perceived constraints. Others control = perceived control over others’ welfare.

^a^Wave 1 ^b^Wave 2 ^c^Wave 3

Supplementary Table 2

*Baseline Descriptive Statistics by Latent Profile*

|  | LC | FC | WC | DS |
| --- | --- | --- | --- | --- |
| Variable | *M* (*SE*) | *M* (*SE*) | *M* (*SE*) | *M* (*SE*) |
| Age^b^ | 55.99 (.520) | 50.45 (.570) | 55.64 (.511) | 57.51 (.618) |
| Sex (% female)^a^ | 65% | 56% | 49% | 63% |
| Race (% white)^a^ | 85% | 86% | 92% | 86% |
| Education^b^ | 6.61 (.116) | 7.09 (.127) | 8.54 (.120) | 7.40 (.126) |
| Income^b^ | 52,033 | 70,552 | 95,231 | 72,557 |
| Episodic memory^b^ | -0.23 (.046) | 0.09 (.051) | 0.09 (.048) | -0.09 (.051) |
| Executive functioning^b^ | -0.16 (.032) | 0.14 (.035) | 0.27 (.030) | -0.04 (.035) |

*Note.* LC = low control profile. WC = work control profile. FC = family control. DS = domain-specific control profile.

^a^Wave 1 ^b^Wave 2

Supplementary Table 3

*ANCOVA F-Table of Profile Differences in 9-Year Cognitive Functioning*

|  | **ΔEpisodic memory** | | | |
| --- | --- | --- | --- | --- |
| Source | *df* | *MS* | *F* | *p* |
| Baseline EM | 1 | 36.46 | 59.61 | < .001 |
| Age | 1 | 140.50 | 229.74 | < .001 |
| Sex (female) | 1 | 59.23 | 96.85 | < .001 |
| Race (minority) | 1 | 3.12 | 5.11 | .024 |
| Education | 1 | 6.03 | 6.03 | .014 |
| Income | 1 | 9.64 | 9.64 | .002 |
| Health status | 1 | 7.39 | 7.39 | .007 |
| Profile | 3 | 0.66 | 1.08 | .356 |
| Error | 2574 | 0.61 |  |  |
|  |  | | | |
|  | **ΔExecutive functioning** | | | |
| Source | *df* | *MS* | *F* | *p* |
| Baseline EM | 1 | 13.23 | 66.89 | < .001 |
| Age | 1 | 43.92 | 220.45 | < .001 |
| Sex (female) | 1 | 0.28 | 1.41 | .235 |
| Race (minority) | 1 | 2.75 | 13.79 | < .001 |
| Education | 1 | 0.81 | 4.08 | .043 |
| Income | 1 | 1.64 | 8.25 | .004 |
| Health status | 1 | 1.14 | 5.71 | .017 |
| Profile | 3 | 0.69 | 3.46 | .016 |
| Error | 2330 | 0.20 |  |  |

*Note*. Outcome measures reflect regressed 9-year changes in episodic memory and executive functioning. Δ = regressed change.

Supplementary Table 4

***Family Control*** *Supplemental Mediation Analyses Predicting 9-Year Changes in Affect (Positive Affect, Negative Affect) and Cognitive Functioning (Episodic Memory, Executive Functioning)*

|  | Episodic memory models | | | | | | |  | Executive functioning models | | | | | | |
| --- | --- | --- | --- | --- | --- | --- | --- | --- | --- | --- | --- | --- | --- | --- | --- |
|  | ΔPA |  | ΔEM |  | ΔNA |  | ΔEM |  | ΔPA |  | ΔEF |  | ΔNA |  | ΔEF |
| Predictors | *b (SE)* |  | *b (SE)* |  | *b (SE)* |  | *b (SE)* |  | *b (SE)* |  | *b (SE)* |  | *b (SE)* |  | *b (SE)* |
| Baseline EM | .00 (.015) |  | -.14 (.019)* |  | -.03 (.011)* |  | -.14 (.019)* |  | - |  | - |  | - |  | - |
| Baseline EF | - |  | - |  | - |  | - |  | -.04 (.025) |  | -.14 (.018)* |  | -.02 (.018) |  | -.15 (.018)* |
| Age | .00 (.001)* |  | -.02 (.002)* |  | -.00 (.001) |  | -.02 (.001)* |  | .00 (.001) |  | -.01 (.001)* |  | -.00 (.001)* |  | -.01 (.001)* |
| Sex (female) | .03 (.028) |  | .33 (.034)* |  | .00 (.020) |  | .34 (.034)* |  | .03 (.027) |  | -.02 (0.020) |  | -.02 (.02) |  | -.02 (.019) |
| Race (minority) | .08 (.040) |  | -.14 (.050)* |  | -.02 (.029) |  | -.12 (.050)* |  | .06 (.043) |  | -.12 (.031)* |  | -.02 (.031) |  | -.13 (.031)* |
| Education | -.02 (.006)* |  | .02 (.007)* |  | .00 (.004) |  | .02 (.007)* |  | -.02 (.006)* |  | .01 (.004)* |  | .00 (.005) |  | .01 (.004)* |
| Income | .00 (.000)* |  | .00 (.000)* |  | .00 (.000)* |  | .00 (.000)* |  | .00 (.000)* |  | .00 (.000)* |  | .00 (.000)* |  | .00 (.000)* |
| Health status | .09 (.015)* |  | .02 (.019) |  | -.04 (.011)* |  | .02 (.019) |  | .09 (.016)* |  | -.02 (.011) |  | -.05 (.011)* |  | .01 (.01) |
| **LC (vs. FC)** | **-.12 (.039)*** |  | **.03 (.048)** |  | **.08 (.028)*** |  | **.02 (.048)** |  | **-.12 (.040)*** |  | **-.06 (.029)*** |  | **-.09 (.029)*** |  | **-.05 (.029)** |
| WC (vs. FC) | .06 (.036) |  | .05 (.045) |  | .00 (.026) |  | .06 (.048) |  | .07 (.037) |  | -.01 (.027) |  | -.01 (.027) |  | -.00 (.027) |
| DS (vs. FC) | -.05 (.038) |  | .03 (.048) |  | .08 (.028)* |  | .04 (.048) |  | -.04 (.040) |  | -.07 (.029)* |  | .09 (.029)* |  | -.07 (.029)* |
| Baseline PA | -.05 (.019)* |  | .06 (.023)* |  | - |  | - |  | -.05 (.020)* |  | -.01 (.014) |  | - |  | - |
| **ΔPA** | **-** |  | **.11 (.026)*** |  | **-** |  | **-** |  | **-** |  | **.07 (.015)*** |  | **-** |  | **-** |
| Baseline NA | - |  | - |  | -.04 (.020)* |  | -.08 (.034)* |  | - |  | - |  | -.05 (.021)* |  | -.04 (.021)* |
| **ΔNA** | **-** |  | **-** |  | **-** |  | **-.15 (.036)*** |  | **-** |  | **-** |  | **-** |  | **-.09 (.021)*** |

*Note.* LC = low control profile. FC = family control. WC = work control profile. DS = domain-specific control profile. PA = positive affect. NA = negative affect. Analyses were conducted with dummy-coded profile variables that reflected low control, work control, and domain-specific control (reference group = family control) and tested whether positive and negative affect mediated the link between ***family control*** and reduced declines in cognitive functioning. Δ = regressed change. Parameter estimates for the main predictors and mediators in our mediation models are shown in **bold** font.

Supplementary Table 5

***Work Control*** *Supplemental Mediation Analyses Predicting Changes in Affect (Positive Affect, Negative Affect) and Cognitive Functioning (Episodic Memory, Executive Functioning)*

|  | Episodic memory models | | | | | |  | | Executive functioning models | | | | | | |
| --- | --- | --- | --- | --- | --- | --- | --- | --- | --- | --- | --- | --- | --- | --- | --- |
|  | ΔPA |  | ΔEM |  | ΔNA |  | ΔEM |  | ΔPA |  | ΔEF |  | ΔNA |  | ΔEF |
| Predictors | *b (SE)* |  | *b (SE)* |  | *b (SE)* |  | *b (SE)* |  | *b (SE)* |  | *b (SE)* |  | *b (SE)* |  | *b (SE)* |
| Baseline EM | .00 (.015) |  | -.14 (.019)^*^ |  | -.03 (.011)^*^ |  | -.14 (.019)^*^ |  | - |  | - |  | - |  | - |
| Baseline EF | - |  | - |  | - |  | - |  | -.04 (.025) |  | -.14 (.018)^*^ |  | -.02 (.018) |  | -.15 (.018)^*^ |
| Age | .00 (.001)^*^ |  | -.02 (.002)^*^ |  | -.00 (.001)^*^ |  | -.02 (.002)^*^ |  | .00 (.001) |  | -.01 (.001)^*^ |  | -.00 (.001)^*^ |  | -.01 (.001)^*^ |
| Sex (female) | .03 (.028) |  | .33 (.034)^*^ |  | .00 (.020) |  | .34 (.034)^*^ |  | .03 (.027) |  | -.02 (.020) |  | -.02 (.020) |  | -.02 (.020) |
| Race (minority) | .08 (.040) |  | -.14 (.050)^*^ |  | -.02 (.029) |  | -.12 (.050)^*^ |  | .06 (.043) |  | -.12 (.031)^*^ |  | -.02 (.031) |  | -.13 (.031)^*^ |
| Education | -.02 (.006)^*^ |  | .02 (.007)^*^ |  | .00 (.004) |  | .02 (.007)^*^ |  | -.02 (.006)^*^ |  | .01 (.004)^*^ |  | .00 (.005) |  | .01 (.004)^*^ |
| Income | .00 (.00)^*^ |  | .00 (.000)^*^ |  | .00 (.000)^*^ |  | .00 (.000)^*^ |  | .00 (.000)^*^ |  | .00 (.000)^*^ |  | .00 (.000)^*^ |  | .00 (.000)^*^ |
| Health status | .09 (.015)^*^ |  | .02 (.019) |  | -.04 (.011)^*^ |  | .02 (.019) |  | .09 (.016)^*^ |  | .02 (.011) |  | -.05 (.011)^*^ |  | .01 (.011) |
| **LC (vs. WC)** | **-.18 (.038)^*^** |  | **-.03 (.048)** |  | **.08 (.028)^*^** |  | **_.04 (.047)** |  | **-.19 (.039)^*^** |  | **-.05 (.028)^*^** |  | **.08 (.029)^*^** |  | **-.05 (.028)** |
| FC (vs. WC) | -.06 (.036) |  | -.05 (.045) |  | -.00 (.026) |  | -.06 (.045) |  | -.07 (.037) |  | .01 (.027) |  | -.01 (.027) |  | .001 (.027) |
| DS (vs. WC) | -.10 (.037)^*^ |  | -.03 (.046) |  | .08 (.027)^*^ |  | -.03 (.046) |  | -.11 (.038)^*^ |  | -.07 (.027)^*^ |  | .08 (.028)^*^ |  | -.07 (.027)^*^ |
| Baseline PA | -.05 (.019)^*^ |  | .06 (.024)^*^ |  | - |  | - |  | -.05 (.012)^*^ |  | -.01 (.014) |  | - |  | - |
| **ΔPA** | **-** |  | **.11 (.026)^*^** |  | **-** |  | **-** |  | **-** |  | **.07 (.015)^*^** |  | **-** |  | **-** |
| Baseline NA | - |  | - |  | -.04 (.020)^*^ |  | -.08 (.035)^*^ |  | - |  | - |  | -.05 (.021)^*^ |  | -.04 (.021)^*^ |
| **ΔNA** | **-** |  | **-** |  | **-** |  | **-.15 (.036)^*^** |  | **-** |  | **-** |  | **-** |  | **-.09 (.012)^*^** |

*Note.* LC = low control profile. WC = work control profile. FC = family control. DS = domain-specific control profile. PA = positive affect. NA = negative affect. Analyses were conducted with dummy-coded profile variables that reflected low control, family control, and domain-specific control (reference group = work control) and tested whether positive and negative affect mediated the link between ***work control*** and reduced declines in cognitive functioning. Δ = regressed change. Parameter estimates for the main predictors and mediators in our mediation models are shown in **bold** font.

Supplementary Table 6

*Tests of Indirect Effects for the Supplemental Mediation Analyses*

| Predictor variable (contrast) | Mediating variable | Outcome variable | Indirect effect^a^ | 95% *CI*s (lower, upper)^b^ |
| --- | --- | --- | --- | --- |
| **Family control models (reference group = family control)** | | |  |  |
| Low control (vs. family control) | ΔPositive affect | ΔEpisodic memory | -.0133^*^ | -.0273, -.0047 |
| Low control (vs. family control) | ΔNegative affect | ΔEpisodic memory | -.0120^*^ | -.0248, -.0040 |
| Low control (vs. family control) | ΔPositive affect | ΔExecutive functioning | -.0086^*^ | -.0175, -.0030 |
| Low control (vs. family control) | ΔNegative affect | ΔExecutive functioning | -.0079^*^ | -.0225, -.0018 |
| **Work control models (reference group = work control)** | | |  |  |
| Low control (vs. work control) | ΔPositive affect | ΔEpisodic memory | -.0193^*^ | -.0348, -.0092 |
| Low control (vs. work control) | ΔNegative affect | ΔEpisodic memory | -.0117^*^ | -.0237, -.0044 |
| Low control (vs. work control) | ΔPositive affect | ΔExecutive functioning | -.0133^*^ | -.0235, -.0067 |
| Low control (vs. work control) | ΔNegative affect | ΔExecutive functioning | -.0069^*^ | -.0197, -.0015 |

*Note.* Mediated profile differences in 9-year episodic memory and executive functioning (indirect effects). Analyses were conducted with two sets of dummy-coded profile variables. The first reflected low control, work control, and domain-specific control (reference group = family control) and tested whether positive and negative affect mediated the link between family control and reduced declines in cognitive functioning. The second reflected low control, family control, and domain-specific control (reference group = work control) and tested whether positive and negative affect mediated the link between work control and reduced declines in cognitive functioning. Δ = regressed change.

aUnstandardized indirect effects are reported for all variables

^b^Confidence intervals are based on 5,000 samples of the unstandardized beta weights.

^*^*p* < .05 based on unstandardized *CI*s (two-tailed tests).

Supplementary Table 7

*Pairwise Comparisons of 9-Year Functional Limitations and Chronic Conditions*

| Outcome measure | LC (1) | FC (2) | WC (3) | DS (4) | Pairwise comparisons |
| --- | --- | --- | --- | --- | --- |
| ΔFunctional limitations | 0.38 | 0.27 | 0.21 | 0.27 | 1 < 2, 3, 4 |
| ΔChronic conditions | 1.26 | 0.79 | 0.74 | 0.83 | 1 < 2, 3, 4 |

*Note.* LC = low control profile. WC = work control profile. FC = family control. DS = domain-specific control profile. Pairwise comparisons involved *t*-tests that contrasted each pair of covariate-adjusted means of functional limitations and chronic conditions.

Covariate-adjusted mean values were adjusted for model covariates that included age, sex, race, education, income, self-reported health status, and autoregressive effects and for (raw) average sample increases of 0.268 units in functional limitations and 0.920 units in chronic conditions. Δ = regressed change.

^*^*p* ≤ .05. ^**^*p* ≤ .01 (two-tailed tests).

Supplementary Table 8

*Hazard Ratios for 18-Year Mortality (Longitudinal Wave 2-3 Sample Who Died Following the Third Interview from 2013-2022 (Total N = 2595))*

| Predictor variables | Hazard ratio | 95% CI |
| --- | --- | --- |
| Age | 1.10^*^ | 1.091, 1.113 |
| Sex (female) | 0.69^*^ | 0.567, 0.833 |
| Race (minority) | 0.68^*^ | 0.485, 0.917 |
| Education | 0.97 | 0.930, 1.007 |
| Income | 0.98^†^ | 0.957, 1.002 |
| Health status | 0.69^*^ | 0.623, 0.763 |
| LC (vs. FC) | 1.32^†^ | 0.991, 1.763 |
| WC (vs. FC) | 1.07 | 0.789, 1.438 |
| DS (vs. FC) | 1.27 | 0.942, 1.709 |
| Log likelihood | -2919 |  |
| Akaike information criterion | 5856 |  |
| *n* (*n* event) | 2595 (442) | |

*Note.* Results of Cox regression model for the longitudinal sample with data from both Wave 2 and Wave 3 who died following the third interview from 2013-2022. Analyses were conducted with dummy-coded profile variables that reflected low control, work control, and domain-specific control (reference group = family control). Income was scaled in $10,000 increments for interpretation.

^†^*p* < .10, ^*^*p* < .05 (two-tailed tests).

Supplementary Table 9

*Hazard Ratios for 18-Year Mortality (Cross-Sectional Wave 2 Sample Who Died Following the Second Interview from 2004-2022 (Total N = 4290))*

| Predictor variables | Hazard ratio | 95% CI |
| --- | --- | --- |
| Age | 1.09^*^ | 1.085, 1.097 |
| Sex (female) | 0.67^*^ | 0.600, 0.758 |
| Race (minority) | 0.98 | 0.842, 1.144 |
| Education | 0.97^*^ | 0.949, 0.996 |
| Income | 0.97^*^ | 0.960, 0.989 |
| Health status | 0.68^*^ | 0.645, 0.726 |
| LC (vs. FC) | 1.23^*^ | 1.046, 1.449 |
| WC (vs. FC) | 0.94 | 0.786, 1.118 |
| DS (vs. FC) | 1.11 | 0.927, 1.333 |
| Log likelihood | -7818 |  |
| Akaike information criterion | 15653 |  |
| *n* (*n* event) | 4290 (1185) | |

*Note.* Results of Cox regression model for the cross-sectional (full) sample with data from Wave 2 who died following the second interview from 2004-2022. Analyses were conducted with dummy-coded profile variables that reflected low control, work control, and domain-specific control (reference group = family control). Income was scaled in $10,000 increments for interpretation.

^†^*p* < .10, ^*^*p* < .05 (two-tailed tests).

Supplementary Figure 1. Elbow plots of the information criterion indices for the latent profiles of domain-specific and domain-general control.

Supplementary Figure 2*.* Results from the *k* = 4 profile model of domain-specific and domain-general perceived control. The low control profile (*n* = 1310) reflected individuals with very low levels of control across all aspects of domain-specific and domain-general control. The family control profile (*n* = 1151) reflected individuals with high levels of control over their family relationships with children and spouses and relatively average levels of control over most other aspects (with above average levels of mastery). The work control profile (*n* = 1474) had higher levels of control over work and finances, lower levels of control over family relationships with children and spouses, and above average levels of mastery and below average levels of control. The high domain-specific control profile (*n* = 860) had high levels of control over all aspects of domain-specific control but had average levels of mastery and constraints.

**Mplus Syntax for the 4-Profile Model**

DATA: FILE = MIDUS PC LPAs Dataset (analyzed MK 5.23.24).csv;

VARIABLE:

NAMES = M2ID

PM_2 CS_2

hlthPC_2 wrkPC_2 fncPC_2 othPC_2 chldPC_2 spsPC_2 ;

IDVARIABLE IS M2ID;

MISSING ARE ALL (-99999) ;

USEVARIABLES = hlthPC_2 wrkPC_2 fncPC_2 othPC_2 chldPC_2

spsPC_2 PM_2 CS_2;

CLASSES = PC (4);

ANALYSIS: TYPE = MIXTURE;

STARTS = 2000 500;

PROCESSORS = 10;

MODEL: %OVERALL%

!estimate variances

hlthPC_2 wrkPC_2 fncPC_2 othPC_2 chldPC_2

spsPC_2 PM_2 CS_2;

!estimate means

[hlthPC_2 wrkPC_2 fncPC_2 othPC_2 chldPC_2

spsPC_2 PM_2 CS_2];

! Specification of the latent factor representing conditional dependence.

F1 BY hlthPC_2* wrkPC_2 fncPC_2 othPC_2 chldPC_2

spsPC_2 PM_2 CS_2;

[F1@0]; F1@1;

%PC#1%

!estimate variances

hlthPC_2 wrkPC_2 fncPC_2 othPC_2 chldPC_2

spsPC_2 PM_2 CS_2;

!estimate means

[hlthPC_2 wrkPC_2 fncPC_2 othPC_2 chldPC_2

spsPC_2 PM_2 CS_2];

%PC#2%

!estimate variances

hlthPC_2 wrkPC_2 fncPC_2 othPC_2 chldPC_2

spsPC_2 PM_2 CS_2;

!estimate means

[hlthPC_2 wrkPC_2 fncPC_2 othPC_2 chldPC_2

spsPC_2 PM_2 CS_2];

%PC#3%

!estimate variances

hlthPC_2 wrkPC_2 fncPC_2 othPC_2 chldPC_2

spsPC_2 PM_2 CS_2;

!estimate means

[hlthPC_2 wrkPC_2 fncPC_2 othPC_2 chldPC_2

spsPC_2 PM_2 CS_2];

%PC#4%

!estimate variances

hlthPC_2 wrkPC_2 fncPC_2 othPC_2 chldPC_2

spsPC_2 PM_2 CS_2;

!estimate means

[hlthPC_2 wrkPC_2 fncPC_2 othPC_2 chldPC_2

spsPC_2 PM_2 CS_2];

Output: sampstat stdyx residual TECH1 TECH13 TECH11 TECH14;
